# Suicides in general hospitals: Meta-analysis of incidence and trends

**DOI:** 10.1177/00048674261441088

**Published:** 2026-04-26

**Authors:** Jack Schrader, Swapnil Sharma, Anthony Azer, Matthew M Large

**Affiliations:** 1 Discipline of Psychiatry & Mental Health, University of New South Wales, Sydney, NSW, Australia; 2 Mindgardens Neuroscience Network, Prince of Wales Hospital, Randwick, NSW, Australia

**Keywords:** Suicide, general hospitals, epidemiology, inpatients

## Abstract

**Objective:**

To synthesise the primary literature reporting the incidence of suicide in general hospitals.

**Method:**

Peer-reviewed papers reporting suicides among the medically and surgically admitted patients of general hospitals were located by searches of MEDLINE, PsycINFO and EMBASE between 1946 and 2025. Random effects meta-analyses were used to estimate the number of suicides per 1,000,000 admissions, the rate of suicide per 100,000 patient years and the proportion of suicides by common suicide methods. Temporal trends were examined with mixed-effects meta-regression.

**Results:**

The pooled number of suicides per 1,000,000 admissions was 16.3 (95% confidence interval = [9.8, 27.0]). The pooled rate of suicide per 100,000 patient-years was 82.7 suicides (95% confidence interval = [49.6, 115.7]). Jumping accounted for 52.1% (95% confidence interval = [39.4, 64.4]) of suicides, and 20.6% (95% confidence interval = [13.1, 30.8]) were by hanging. The number of suicides per admission declined over time (point estimate of slope = −0.039, standard error = 0.01, *p* < 0.0004) to 4.7 suicides per 1,000,000 admissions (95% confidence interval = [1.7, 12.9], *I*^2^ = 97) in nine studies published after 2010. The rate of suicide per patient year was unchanged over 60 years of primary research (slope ⩽ −0.0001, standard error = 0.0001, *p* = 0.97).

**Conclusion:**

The rate of suicide in general hospital inpatients is an order of magnitude higher than the global suicide rate. While general hospital suicide is a critical patient safety concern, the stability in suicide rates over time highlights the persistent difficulty of suicide prevention in this setting.

The medical and surgical wards of general hospitals are places of care, diagnosis, treatment and healing. Tragically, they are also the setting of some suicides (Ballard et al., 2008; Burns and Sher, 2025). All suicides entail the loss of years of life and have severe effects on families and friends (Cerel et al., 2008). Inpatient suicides also impact on treating teams (Fairman et al., 2014; Malik et al., 2022) and carry a burden of legal liability (Cantor and McDermott, 1994). The incidence of suicide among current psychiatric inpatients has been the subject of at least one meta-analysis (Walsh et al., 2015). In contrast, the incidence of suicide in general medical and surgical settings is under-researched and has not been estimated by meta-analysis, despite studies from the United States, Australia and Japan finding that between a quarter and a half of all inpatient suicides are by people admitted to general medical and surgical wards (Inoue et al., 2017; Sweeting et al., 2023; Williams et al., 2018). A recent review (Burns and Sher, 2025) highlighted the limited nature of research on inpatient suicides outside the psychiatric setting and emphasised the underrecognised nature of general hospital suicides. A 2008 systematic review of suicides in general hospitals was unable to quantify the incidence of suicide and was limited by what the authors described as selection bias and inconsistency of reporting (Ballard et al., 2008). Since 2008, new studies of general hospital suicide have been published (Ang, 2018; Rucco et al., 2023; Shekunov et al., 2013; Sweeting et al., 2023; Tan et al., 2018; Tseng et al., 2011), creating the opportunity for a meta-analysis of the number of suicides per general hospital admission and the rate of suicides per bed year. Furthermore, the epidemiology of suicide in general hospitals may also have changed over time. Recent decades have seen a focus on hospital safety (Bates and Singh, 2018), dramatic falls in average length of hospital stay (Clarke and Rosen, 2001) and the development of consultation liaison psychiatry (Ali et al., 2006). At the same time, there have been changes in hospital design that reduce the opportunity for suicide by jumping (White et al., 1995). Quantification of the number and rate of general hospital suicides and possible trends over time might reflect the effects of these changes and could inform planning of future hospital services and infrastructure.

Accordingly, the primary aim of this review was to fill a knowledge gap by deriving pooled estimates of the number per million admissions and rate per 100,000 patient-years of suicide among currently admitted general hospital inpatients. The secondary aim was to examine temporal changes in the incidence and methods of suicide in this setting.

We report a meta-analysis estimating (1) the number of suicides per 1,000,000 general hospital admissions, (2) the rate of suicide per 100,000 general hospital patient-years and (3) the proportion of suicides by common suicide methods in this setting. We hypothesised that more recent studies would report a lower incidence of suicide and a smaller proportion of suicides by jumping.

## Method

We conducted a meta-analysis of the incidence of suicides among currently admitted medical and surgical patients in general hospitals according to Preferred Reporting Items for Systematic reviews and Meta-Analyses (PRISMA) guidelines (Page et al., 2021) and registered with INPLASY (Canellas et al., 2023) (DOI 10.37766/inplasy2025.7.006). A detailed study protocol was not published but the methods used (including the analysis of mortality per admission and per bed-patient year, and the meta-analytic statistical methods), mirrored analogous meta-analyses of suicide rates (Walsh et al., 2015) and cause-specific mortality (Angel-Scott et al., 2025) among current psychiatric inpatients.

### Definitions

General hospital suicides were defined as intentional acts with a fatal outcome among the currently admitted general hospital inpatients. General hospital inpatients were defined as patients admitted to medical and surgical wards, emergency departments and intensive care wards. Mental health and toxicology wards were defined as psychiatric settings and suicides in these environments were excluded from the study. General hospital suicides, therefore, included suicides of patients whose primary admission was to medical or surgical services, irrespective of any co-occurring mental illness or whether the suicide occurred on the ward, elsewhere on the hospital grounds, or while the patient was on any sort of leave according to convention (Oehmichen and Staak, 1988). Reports of deaths resulting from actions before admission and suicides after hospital discharge were excluded from the study.

### Search strategy

Searches for papers reporting the incidence of suicide were conducted as part of a broader systematic review of suicidal thoughts and behaviours in general hospitals. English Language publications indexed in EMBASE, PsycINFO or MEDLINE between 1946 and 29 December 2025, were identified with the terms: (suicide* or self-harm or self harm) and Inpatient or In-patient) and (Somatic or medical or surgical or general hospital).af or (Suicidal ideation AND General Hospital and Suicide and General Hospital).mp.). A second set of searches was completed for titles in PubMed from inception until 29 December 2025, including the terms ((suicid*) AND (hospital* OR inpatient OR in-patient OR admitt*).

### Study selection

Two reviewers (J.S. and M.L.) independently reviewed titles and abstracts based on likelihood of meeting the inclusion criteria. Subsequently, J.S. and M.L. examined the resulting papers in full texts for inclusion and exclusion, with differences in study selection resolved by a joint examination and consensus. The electronic searches were supplemented by hand searching the reference lists of review articles (Ballard et al., 2008; Bostwick and Rackley, 2007; Burns and Sher, 2025; Kellner et al., 1985) and all of the examined full texts.

Studies were included if they reported on suicides among a consecutive series of admitted general hospital patients. Individual case reports and non-consecutive cases were excluded. Studies reporting on deliberate self-harm or suicide attempts, but no suicide deaths, were included in the analysis with zero values. There were no other limits on study design. Studies were excluded if they reported on suicide and suicide attempts such that they could not be separated, reported on general hospital and psychiatric inpatient suicides such that they could not be separated, included deaths resulting from suicidal behaviour before the admission or reported deaths resulting from non-intentional self-harm. In the event of two studies reporting on the same suicides, we included the numerically larger study.

### Data extraction

J.S. and M.L. independently extracted the data, with any differences resolved by further examination and consensus by S.S. and M.L.

Numerator data included the total number of suicides, and the number of suicides by jumping, hanging, overdose and firearm.

The denominator of the number of total admissions among which the suicides occurred was collected to calculate the number of suicides per 1,000,000 admissions. This denominator allowed for an estimate of the probability of an admission ending in a suicide and the risk faced by each patient at the point of admission.

The denominator of the number of patient-years (or the number of bed years when the number of patient-years was not reported) was collected to calculate the rate of suicide per 100,000 patient-years. This rate is an estimate of the likelihood of a suicide in a particular bed per year, represents the risk faced by a hospital of a suicide in any given period and is directly comparable to the rate of suicide per 100,000 person-years as conventionally reported for suicides in the community.

The denominator of the total number of suicides was collected to estimate the proportion of suicides according to methods.

The mid-year of suicide data-ascertainment in each primary study was determined by dividing the sum of the first and last year of data collection by two and was used as moderator data to examine secular trends in the number of suicides per admission and suicide rate per patient-year.

### Strength of reporting

A four-item strength of reporting scale derived from the Newcastle–Ottawa Strength of Reporting Scale (Wells et al., 2000) was used to assess each primary study. The scale included one item appraising representativeness of the patient sample (Item 1: Recruitment of admissions from defined geographic catchment area), one item appraising measurement of exposure (Item 2: Reporting the number of admissions and number of patient-years) and two items appraising ascertainment of outcome (Item 3: Utilisation of external mortality databases to determine death by suicide and Item 4: Reporting suicide methods).

### Meta-analysis and meta-regression

We performed a meta-analysis because a simple summation of suicides with respect to the aggregate number of person-years or admissions is not appropriate for synthesising results from multiple studies, because it treats the data as if they were derived from a single homogeneous cohort. Meta-analytic methods instead analyse study-level rates and weight them according to their variance, allowing both within-study uncertainty and between-study heterogeneity to be incorporated into the pooled estimate and its confidence intervals (CIs) (Barendregt et al., 2013).

A random effects model using the DerSimonian & Laird method (DerSimonian and Laird, 1986) was chosen for all analyses because of an a priori assumption about differences in the settings and patient populations examined in primary studies conducted internationally over the time spanned by the research reviewed.

After the analysis, suicides per admission were converted to suicides per 1,000,000 admissions, and the suicides per patient-year were converted to the rate of suicide per 100,000 patient years, to aid clarity and to assist the reader in differentiating between the analyses.

The expected range of the estimated pooled value was reported using 95% CIs, and 95% prediction intervals (PIs) were calculated to estimate the probable range of any future study.

For each analysis, heterogeneity was estimated with an *I*^2^ statistic. Publication bias was assessed for each analysis by inspection of funnel plots and Egger’s regression. If Egger’s regression indicated publication bias (*p* ⩽ 0.05), Duval and Tweedie’s trim and fill method was used to estimate the effect of possible publication bias on the pooled estimate. Potential outliers were identified through inspection of the forest plot and confirmed using studentised residuals; samples with absolute studentised residuals of >3 were regarded as outliers. A sensitivity analysis assessed the impact of outliers on the pooled effect size.

A mixed-effects regression model (restricted maximum likelihood, *z*-distribution) was used to explore the association between the moderator of mid-year of primary study data ascertainment and between-study heterogeneity. This allowed an examination of secular trends in the number of suicides per admission, the rate of suicide and the proportion of deaths according to suicide methods. All analyses were conducted using Comprehensive Meta-analysis V4 (Borenstein et al., 2022).

## Results

### Search results

**Figure 1.**
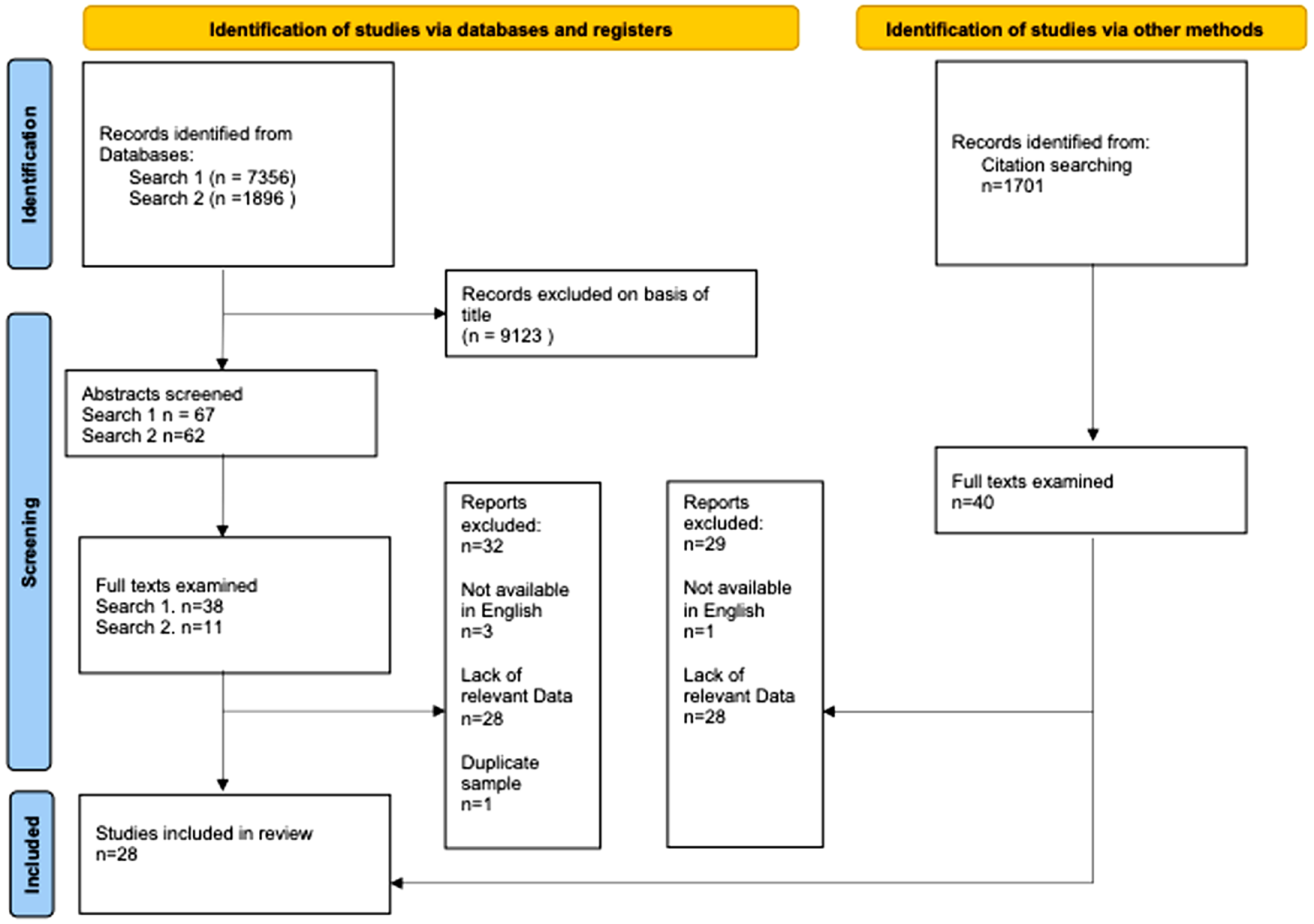
Flowchart of searches.

The first database search and hand searching of reference lists yielded 9057 titles, and the second search identified 1896 titles. No additional papers were identified by the second electronic search (Figure 1). After examination of 89 full texts, 29 papers met the inclusion criteria before one was excluded (Pollack, 1957) because it described suicides also reported in an included paper (Ripley, 1979), leaving 28 included papers. These 28 papers (Altinoz et al., 2019; Ang, 2018; Brown and Pisetsky, 1960; Cheng et al., 2009; Copeland, 1986; Farberow et al., 1971; Friedman and Cancellieri, 1958; Glickman, 1980; Ho and Tay, 2004; Hung et al., 2000; Inoue et al., 2017; Mills et al., 2021; Mills et al., 2014; Petrovsky, 1967; Reich and Kelly, 1976; Ripley, 1979; Rucco et al., 2023; Shapiro and Waltzer, 1980; Shekunov et al., 2013; Stoller and Estess 1960; Suominen et al., 2002; Sweeting et al., 2023; Tan et al., 2018; Thurston, 1957; Tseng et al., 2011; Wan et al., 2020; White et al., 1995; Williams et al., 2018) reported total of 972 general hospitals suicides (median per study = 16.5, interquartile range = 24.5) (Table 1). Three papers reported two studies at different hospitals or types of general hospital settings.

**Table 1.** Table of included studies.

Study name	Research setting and period of data ascertainment	Determination of outcome	Suicides, *N*	Outcomes
Altinoz et al. (2019)	Eskisehir Osmangazi University Hospital, Turkey. 2003–2017	Police records	2	Methods
Ang (2018)	Tan Tock Seng Hospital, Singapore. 2000–2015	Sentinel event reporting	20	Methods Admissions Rates
Brown and Pisetsky (1960)	Bronx Veteran’s Administration Hospital, USA. 1947–1958	Unclear	16	Methods Admissions
Copeland (1986)	Medical examiner Dade County 1980–1984	Medical examinations	2	Methods
Cheng et al. (2009)	Far Eastern Memorial Hospital, Taiwan. 1995–2004	Adverse event reports	17	Admissions Rates
Farberow et al. (1971)	Multiple US Veterans Administration Hospitals, USA. 1959–1966	Reporting to Central Research Unit	283	Methods Admissions
Friedman and Cancellieri (1958)	Fordham Hospital, NY USA. 1954–1957	Chart Review	2	Methods Admissions
Glickman (1980)	Kings County Hospital, New York, USA. 1963–1978	Unclear	22	Methods Admissions Rates
Ho and Tay (2004)	26 hospitals in Hong Kong, China. 2000–2002	Review of reports to hospital management	34	Methods Admissions Rates
Hung et al. (2000)	Chang Gung Memorial Hospital, Taiwan. 1988–1997	Daily incident reports	15	Methods Admissions Rates
Inoue et al. (2017)	Survey of hospitals, Japan. 2012–2015	Reports of suicides from survey respondent hospitals	131	Rates methods
Mills et al. (2014)	Veterans’ Health Administration hospitals, USA. 1999–2012	Root Cause Analysis Reports	5	Admissions Methods
Mills et al. (2021)	Veterans’ Health Administration hospitals, USA. 1999–2018	Root Cause Analysis Reports	17	Admissions Methods
Petrovsky (1967)	Launceston Hospital, Australia. 1954–1966	Unclear	4	Admissions Methods
Reich and Kelly (1976)	Peter Bent Brigham Hospital, USA. 1967–1973	Incident reports, records of consultations, discharge diagnoses and recollections of key personnel	0	Rates Admissions
Ripley (1979)	General Hospital, USA. 1965–1975	Unclear	14	Methods
Rucco et al. (2023)	Milan institute of forensic medicine Italy. 1993–2020	Review of autopsy reports	63	Methods
Shapiro and Waltzer (1980)	Queens Hospital Centre, New York, USA. 1965–1979	Unclear	12	Rates Admissions Methods
Shekunov et al. (2013)	Mayo Clinic Rochester, New York, USA. 1998–2010	Adverse event and sentinel event reports	1	Admissions
Stoller and Estess (1960)	Two US general Hospitals. 1946–1955	Hospital medical records	33	Admissions Methods
Suominen et al. (2002)	General Hospitals Finland. 1987–1988	Assessed by detailed investigations as part of psychological autopsy	26	Methods
Sweeting et al. (2023)	General hospital admissions in Australia. 2009–2018	National Coronial Information System records	87	Methods
Tan et al. (2018)	Tongji Medical College, Hubai, China. 2008–2017	Patient safety data	24	Methods Admissions Rates
Thurston (1957)	Hospitals in the West London Coroners District 1956	Coroner’s data	6	Methods Admissions Rates
Tseng et al. (2011)	Teaching general hospital in Taiwan. 1995–2004	Adverse event reports	27	Rates
Wan et al. (2020)	48 General Hospitals in Hubei, China. 2015–2017	Epidemiological surveys provided to hospitals	89	Admissions
White et al. (1995)	Royal Prince Alfred Hospital, NSW, Australia. 1980–1992	Computerised search and retrieval of relevant medical records	8	Rates Admissions Methods
Williams et al. (2018)	Hospitals US states. 2010–2017	National Violent Death Reporting System	12	Rates Admissions

Twenty papers reporting the numbers of suicides and admissions were included in the estimates of suicides per 1,000,000 admissions (with a total of 23 studies because three papers reported on two data sets [Ang, 2018; Mills et al., 2021; Stoller and Estess, 1960], see Supplemental Material (SM) 1. Data used in the meta-analysis).

Thirteen papers reporting the number of suicides and patient (or bed) years were included in the estimates of the rate of suicide per 100,000 patient-years (with a total of 14 studies as one study reported suicides in two separate periods [Ang, 2018], see SM 1. Data used in the meta-analysis).

Twenty-two papers were included in the analysis of the proportion of suicides by method (with a total of 25 studies, because three papers reported on two data sets [Ang, 2018; Mills et al., 2021; Stoller and Estess, 1960], see SM 1. Data used in the meta-analysis).

The 28 papers had a mean strength of reporting score of 2.1 and a median score of 2 (see SM 2, Strength of Reporting Scores). A summary of the results can be found in Table 2, and the results according to suicides per admission, suicides per patient year and suicides according to method are presented in detail below.

**Table 2.** Summary of results of meta-analysis of general hospital suicide.

	No. of studies	No. of suicides	Denominator	Pooled estimate	Lower limit	Upper limit	*I* ^2^	Temporal trend
Meta-analysis of suicides per admission								
Suicides per million admissions	23	610	56,991,044 admissions	16.3	9.8	27.0	97	Falling
Meta-analysis of suicides per patient year								
Suicides per 100,000 patient-years	14	327	1,016,217 patient-years	82.7	49.6	115.7	96	Stable
Meta-analysis of common suicide methods								
Percentage of suicides by jumping	25	365	820 suicides	52.1%	39.4%	64.4%	83	Non-significant fall
Percentage of suicides by hanging		196		20.6%	13.1%	30.8%	81	Increasing
Percentage of suicides by sharp object		77		11.2%	6.8%	17.8%	65	Non-significant fall
Percentage of suicides by poisoning		46		7.9%	5.8%	10.7%	4	Stable

### Meta-analysis of general hospital suicides per 1,000,000 admissions

A pooled estimate of suicide of 16.3 suicides per million general hospital admissions (95% CI = [9.8, 27.0]; PI = 1.6–70.6), *I*^2^ = 97) was derived from 610 suicides among 56,991,044 admissions. This equates to one suicide per 61,276 admissions. Most studies were outside the funnel plot (SM 3.1). Egger’s regression suggested publication bias (intercept −3.8, standard error (SE) = 1.6, *p* = 0.03); however, Tweedie and Duval’s trim and fill did not identify any missing studies on either side of the mean. One study was found to be an outlier with a residual of >3 (Williams et al., 2018). The removal of this study increased the pooled estimate to 20 suicides per million admissions (95% CI = [13.1, 30.6]. We did not regard the statistical outlier status of the Williams study as sufficient reason to exclude it from the analysis because it was a large, well conducted and recent study sampling important US data sets.

**Figure 2.**
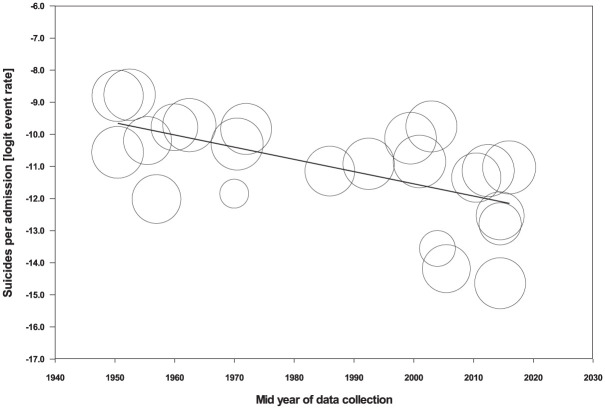
Meta-regression of suicides per admission by mid-year of suicides.

On meta-regression, there was a significant fall in the number of suicides per admission over time (point estimate of slope = −0.039, SE = 0.01, *p* < 0.0004) (Figure 2). By way of illustration, nine studies published after 2010 had a pooled 4.7 suicides per million admissions (95% CI = [1.7, 12.9], *I*^2^ = 97), equivalent to one suicide per 213,229 admissions.

### Meta-analysis of general hospital suicides per 100,000 patient-years

**Figure 3.**
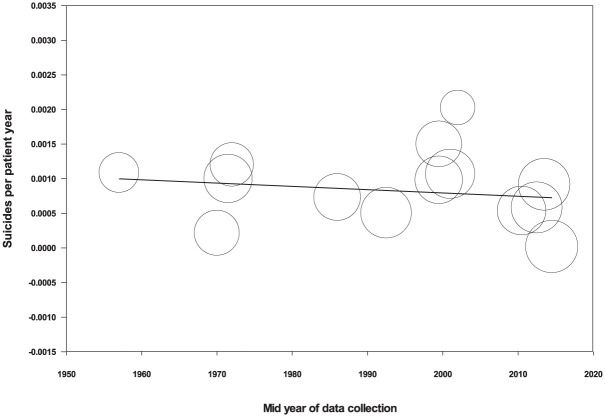
Meta-regression of suicides per patient-year by mid-year of suicides.

A pooled estimate of the general hospital inpatient suicide rate was 82.7 suicides 100,000 patient-years (95% CI = [49.6, 115.7]; PI = 48–200, *I*^2^ = 96) which was derived from 327 suicides occurring during 1,016,217 patient-years. The Forrest Plot showed one outlying study to the left of the mean with a high suicide rate (SM 3.2). Egger’s regression suggested publication bias (intercept = 4.26, SE = 0.73, *p* = 0.0001) with bias towards studies with a higher suicide rate. Duval and Tweedie’s trim and fill method identified one hypothetical missing study to the left of the mean associated with a downwards adjustment of the estimate to 68 suicides per 100,000 patient-years (95% CI = [38, 98]). No study was identified as an outlier by examination of residuals. There was no significant change in the rate of suicide per patient year over time (point estimate of slope ⩽−0.0001, standard SE = 0.0001, *p* = 0.97) (Figure 3).

### Meta-analysis of methods of general hospital suicides

The pooled percentage of suicide by jumping was 52.1% (95% CI = [39.4, 64.4], *I*^2^ = 83). There was no evidence of publication bias detected by examination of the funnel plots (SM 3.3) or with Egger’s regression (intercept = 1.01, SE = 0.69, *p* = 0.16). A possible fall in the proportion of suicides by jumping over time (point estimate of slope ⩽−.016, SE = 0.014, *p* = 0.25) was not statistically significant. The pooled percentage of suicide by hanging was 20.6% (95% CI = [13.1, 30.8], *I*^2^ = 81) with a significant increase in proportion over time (point estimate of slope = 0.025, SE = 0.01, *p* = 0.02). The pooled percentage of suicide by sharp objects was 11.2%, (95% CI = [6.8, 17.8], *I*^2^ = 65) with a non-significant fall over time (point estimate of slope = −0.017, SE = 0.011, *p* = 0.14). The pooled percentage of suicide by poisoning was 7.9% (95% CI = [5.8, 10.7], *I*^2^ = 4.0) without a change over time (point estimate of slope = 0.001, SE = 0.007, *p* = 0.89). In total, 20.0% (95% CI = [13.5, 29.2]) of suicides were by firearm among five studies that reported any deaths by this method.

## Discussion

This study fills a knowledge gap by providing the first meta-analytic synthesis of the number of suicides per million admissions, the rate of suicide per 100,000 patient-years, and methods of suicide by currently admitted general hospital patients. The study used systematic methods to source primary studies spanning decades of international research. The pooled estimate of the rate of general hospital suicides was 82.7 per 100,000 patient-years, a rate that is almost 10 times higher than the current global suicide rate of 9.0 per 100,000 population (World Health Organization [WHO], 2021). This elevated rate underscores the vulnerability to suicide of hospital inpatients admitted for non-psychiatric reasons.

Despite concerted efforts to improve patient safety in hospitals, we were not able, with our sample of studies, to demonstrate decline in the time-adjusted suicide rate. The pooled suicide rate from the 1970s was statistically similar to that in the 2010s. This is a sobering finding that suggests that the intrinsic risk of suicide among hospitalised patients has remained high and relatively constant.

On the contrary, the study found a significant decline in the number of suicides per 1,000,000 admissions. This apparent contradiction of significant decrease in the probability of suicide per admission and the stability of the rate of suicides per 100,000 patient-years over time can be explained by reductions in lengths of general hospital admissions and a reduced time at risk in more recent studies (Golinelli et al., 2023; Reid et al., 2023). It follows, therefore, that there has been little progress in reducing the number of suicides that a general hospital can expect over any given time frame. Our findings do not support the hypothesis that the effects of improved environmental safety and higher standards of medical and psychiatric care have reduced suicide rates in the general hospital.

The findings highlight that jumping from a height and to a lesser extent, hanging are the two predominant methods of inpatient suicide in general hospital settings, together accounting for around three-quarters of cases. This pattern differs somewhat from psychiatric units where hanging tends to dominate (Hunt et al., 2012).

The limitations of this meta-analysis need to be emphasised. First, the modest number of studies, some with small sample sizes, should temper confidence in our estimates, and encourage a future updated meta-analysis when more data become available. The lack of robustness to the set of available studies is illustrated by the 20% increase in the pooled rate of suicides per million admissions associated with the exclusion of a single study with a low, but statistically outlying number of suicides per admission (Williams et al., 2018). Furthermore, the high statistical heterogeneity was not explained by the moderator variable examined of time of publication. This high but unexplained heterogeneity is likely to be the result of differences in hospital and ward types (including medical and surgical wards, emergency departments and intensive care units), patient mix according to age, sex and diagnosis, and safety cultures that were not reported in the primary literature. The modest number of studies also meant that we could not robustly analyse differences by country, or healthcare system.

Second, the estimate of the crucial metric of the suicide rate per patient year was further limited because most of the included studies did not report sufficient information to calculate the total number of patient-years. The paucity of data and the heterogeneity of the estimates, particularly of the suicide rate, mean the results should be accepted cautiously and provisionally and should not be used administratively as safety benchmarks.

Third, we were unable to examine individual risk factors in this meta-analysis. Questions such as which patients (by diagnosis or other characteristics) are most at risk of suicide during a general hospital admission were beyond our scope, because the primary studies rarely included comparison groups or detailed risk factor analysis. Future primary research could use large modern administrative data sets to investigate risk predictors such as demographic factors, medical diagnoses, somatic treatments and even psychiatric consultations.

Fourth, there was a potential publication bias and data availability bias. Hospitals or regions that experienced very few or zero inpatient suicides would be unlikely to publish those results, whereas those with notable clusters might be more inclined to report. This could inflate the pooled incidence. We were able to include one study that reported suicide attempts without any fatalities (Reich and Kelly, 1976) but could not estimate the extent or impact of this form of publication bias. We did attempt to assess and adjust for publication bias; Egger’s test indicated some asymmetry for the per-admission outcome, but trim-and-fill did not change the estimate. A trim-and-fill analysis of suicides per 100,000 patient years resulted in an 18% reduction in the pooled estimate. This provides reassurance that our results are not greatly overestimated. Nonetheless, under-reporting remains a concern, particularly because most hospitals, regions and countries have no reports of general hospital suicide rates.

Fifth, the use of inconsistent terminology and definitions in the primary studies might also have been a limitation. We took care to apply a consistent definition (including only currently admitted patients and excluding those in psychiatry and toxicology wards), but in practice, some studies might have inadvertently included some cases that others would exclude (e.g. a patient who absconded and died off-site might or might not be counted, depending on the study). Most studies did count suicides on hospital grounds or on approved leave passes, as included by our definition, but not all studies were explicit about their handling of that scenario.

Sixth, our proxy of bed years for patient-years assumes that each hospital had 100% bed occupancy. A limitation of the primary literature was that the number of actual patient-years was rarely reported. The use of this proxy likely overestimated bed days and biased the results towards lower suicide rates than may have occurred.

Finally, we had no way of assessing the extent of post-discharge suicide, that at least in theory, could be a consequence of events in hospital.

With the above caveats, the pooled estimate of the rate of general hospital suicides is about half the meta-analytically derived estimate of suicide rates in psychiatric hospitals (Walsh et al., 2015) and is similar to those reported in prisons (Mundt et al., 2024) and immigration detention centres (Erfani et al., 2021). This result is surprising, given the likely population and environmental differences between these settings, and the availability of prompt psychiatric consultation and care in most general hospitals.

This is not to suggest that the impact of suicides should only be considered in the light of their frequency. Suicides in institutions might be particularly disturbing to family and friends who assume that hospitals are places of safety. Suicides in general hospital settings might also have a greater impact on clinicians than suicides in psychiatric care because staff lack familiarity with psychiatric presentations and suicidality. There is emerging evidence of the toll patient suicides exact on the psychiatric workforce (Gibbons et al., 2019; Malik et al., 2022; Sandford et al., 2021) but the impact of suicide on generalists is less well described.

The contrast between significant reduction in the suicides per 1,000,000 admissions and the stability of the rate of suicide per 100,000 patient-years provides a previously unavailable insight into the unexpected ways in which the phenomenon of suicide in the general hospital setting has changed over time. The included primary research was conducted over the same period of the development and growth of consultation liaison psychiatry (Schwab, 1989) and numerous changes in hospital design and the nature of medical and surgical admissions (Golinelli et al., 2023). The sustained elevation of the rate of suicide per patient year in general hospitals should undermine any complacency around suicide in general hospitals resulting from the fall in suicides per admission.

The lack of change in suicide rates over time and the comparability of the estimates with custodial settings reinforce the need to enhance our current approaches to patient safety in the general hospital. This persistence of the problem of inpatient suicide in the face of advances of modern medical care underlines the challenges inherent in considering the phenomena through the perspective of a biomedical model. The antecedents of suicidal events are so complex that addressing each factor in isolation through a reductionist approach is unlikely to yield effective results (Fazel and Runeson, 2020). Considering the multifactorial effects of suicides on the many stakeholders of a general hospital, it follows that approaches towards addressing their prevalence and harms should be holistic in nature, with consideration of the biological, psychological, social and systemic factors underlying a general hospital patient’s condition.

The analysis of suicide methods found jumping to be the most common method, followed by hanging. In most communities, suicide by jumping is a rare form of suicide. Jumping might be the predominant method of suicide in hospitals because it involves little planning, is highly lethal and may not be prevented by the intervention of others, even in a hospital (Gunnell and Nowers, 1997). The increasing proportion of suicides by hanging might be explained by an increased difficulty of access to places to jump in modern hospitals, combined with the known difficulty of reducing ligature points in medical and surgical settings (Gunnell et al., 2005). Patient safety is a concept that is increasingly employed in other areas of harm-reduction in the hospital environment (Brambilla et al., 2019), including in accidental falls prevention (Piatkowski et al., 2021). Intentional jumping and hanging warrant similar consideration. This may include installing secure window restrictors, limiting access to rooftops and high balconies, enclosing stairwells or removing potential anchor points for hanging. Such considerations would be consistent with minimisation of environmental risks in general hospitals as required in recently published national safety and quality standards (Australian Commission on Safety and Quality in Health Care, 2018).

Staff training and awareness can empower staff to identify people in distress and initiate psychiatric consultations and safety measures. These safety measures should include increased awareness and possibly auditing of jumping and hanging points, and increased monitoring and observation of patients identified as experiencing suicidal thoughts and behaviours. Universal screening of all patients for suicidality has been recommended but remains controversial because while it can increase detection of suicidal ideation, its impact on reducing completed suicides in general hospital populations is unproven and limited by a high false-positive rate (Large, 2013). Nonetheless, while acknowledging technical difficulties in its implementation, given the absence of improvement in suicide rates in general hospitals over the decades of researched reviewed here despite sweeping advances in psychiatric and medical care over this time, we agree with Burns and Sher (2025) that screening of all general hospital inpatients might prove to be a reasonable strategy to attempt to address the persistence of suicide in this setting. Screening alone, without associated interventions, is unlikely to be helpful and would need to be accompanied by a commensurate increase in access to consultation liaison psychiatry services.

For health systems, our results indicate that shorter hospital stays have reduced the number of suicides per admission but not the rate per patient year. One interpretation of this is that the risk may have been shifted elsewhere into the post-discharge period. This underscores a need for strong transitional care for at-risk patients: when a patient with suicidal tendencies is discharged from a general hospital, there must be vigilant follow-up, including with the involvement of mental health services, safety planning and family education.

In future, more routine data collection and improved transparency about inpatient suicides in general hospitals would allow better evaluation of prevention strategies. For example, if universal screening for suicidal ideation upon admission were implemented widely, one could study its impact – but that requires good data on baseline incidence and outcomes. Our work establishes a baseline that could form a basis of comparison in future studies or audits.

## Conclusion

Hospitals are a globally prevalent and essential setting for medical care, but the phenomenon of general hospital suicide is under-researched. A more robust assessment of this aspect of hospital safety will require wider reporting of general hospital suicide rates.

## Supplemental Material

sj-docx-2-anp-10.1177_00048674261441088 – Supplemental material for Suicides in general hospitals: Meta-analysis of incidence and trends Supplemental material, sj-docx-2-anp-10.1177_00048674261441088 for Suicides in general hospitals: Meta-analysis of incidence and trends by Jack Schrader, Swapnil Sharma, Anthony Azer and Matthew M Large in Australian & New Zealand Journal of Psychiatry

sj-docx-3-anp-10.1177_00048674261441088 – Supplemental material for Suicides in general hospitals: Meta-analysis of incidence and trends Supplemental material, sj-docx-3-anp-10.1177_00048674261441088 for Suicides in general hospitals: Meta-analysis of incidence and trends by Jack Schrader, Swapnil Sharma, Anthony Azer and Matthew M Large in Australian & New Zealand Journal of Psychiatry

sj-xlsx-1-anp-10.1177_00048674261441088 – Supplemental material for Suicides in general hospitals: Meta-analysis of incidence and trends Supplemental material, sj-xlsx-1-anp-10.1177_00048674261441088 for Suicides in general hospitals: Meta-analysis of incidence and trends by Jack Schrader, Swapnil Sharma, Anthony Azer and Matthew M Large in Australian & New Zealand Journal of Psychiatry
