# Supplementary material for: Suicides in general hospitals: Meta-analysis of incidence and trends
Source: Aust N Z J Psychiatry. 2026 Apr 26;60(7):687–98. doi: 10.1177/00048674261441088 (PMC13291405; doi:10.1177/00048674261441088)
Supplement: sj-docx-2-anp-10.1177_00048674261441088 – Supplemental material for Suicides in general hospitals: Meta-analysis of incidence and trends [file sj-docx-2-anp-10.1177_00048674261441088.docx]

**Supplementary Materials 2. Strength of reporting of included studies**

| Study | 1 | 2 | 3 | 4 | Total |
| --- | --- | --- | --- | --- | --- |
| Ang 2018 | ● | ● | ● |  | 3 |
| Altinoz et al 2019 | ● |  | ● | ● | 3 |
| Brown and Pisetsky 1960 | ● |  | ● |  | 2 |
| Cheng et al 2009 | ● | ● |  |  | 2 |
| Copeland 1986 | ● |  | ● | ● | 3 |
| Farebrow et al 1971 |  |  | ● |  | 1 |
| Glickman 1980 | ● | ● | ● |  | 3 |
| Ho et al. 2004 | ● | ● | ● |  | 3 |
| Hung et al. 2000 | ● | ● | ● |  | 3 |
| Inoue et al. 2017 |  |  | ● |  | 1 |
| Mills et al 2014 |  |  | ● |  | 1 |
| Mills et al 2021 |  |  | ● |  | 1 |
| Petrovsky 1967 | ● |  | ● |  | 2 |
| Reich and Kelly 1976 | ● | ● |  |  | 2 |
| Ripley 1979 |  |  | ● |  | 1 |
| Rucco et al 2023 | ● |  | ● | ● | 3 |
| Shapiro and Waltzer 1980 | ● | ● | ● |  | 3 |
| Shekunov et al 2013 | ● |  |  |  | 1 |
| Stoller and Estess 1960 | ● |  | ● |  | 2 |
| Suominen et al 2002 | ● |  | ● |  | 2 |
| Sweeting et al 2023 | ● |  | ● | ● | 3 |
| Tan et al 2018 |  | ● | ● |  | 2 |
| Thurston 1957 | ● | ● | ● | ● | 4 |
| Tseng et al 2011 | ● |  |  |  | 1 |
| Wan et al 2020 | ● |  |  |  | 1 |
| Wender 1958 | ● |  | ● |  | 2 |
| White et al 1995 | ● | ● | ● |  | 2 |
| Williams et al 2018 |  | ● |  |  | 1 |

● = item satisfied criterion and scored 1

**Strength of reporting scale definition**

**Item 1:** *Population based.* Score 1 if the cases were recruited from a defined geographic area.

**Item 2:** *Score 1 if both number of beds and number of admissions were recorded.*

**Item 3:** *Inclusion of suicide methods. Score 1 if methods of suicide were listed with recorded deaths.*

**Item 4***: Suicide ascertainment*. Score 1 if a mortality database was used to ascertain death.

Score 0 if any of the above items are not clearly present
