# Supplementary material for: Suicides in general hospitals: Meta-analysis of incidence and trends
Source: Aust N Z J Psychiatry. 2026 Apr 26;60(7):687–98. doi: 10.1177/00048674261441088 (PMC13291405; doi:10.1177/00048674261441088)
Supplement: sj-docx-3-anp-10.1177_00048674261441088 – Supplemental material for Suicides in general hospitals: Meta-analysis of incidence and trends [file sj-docx-3-anp-10.1177_00048674261441088.docx]

| SM 3. Funnel Plots |
| --- |
|  |
| SM 3.1 Funnel Plot of general hospital suicides per admission. |
|  |
| SM 3.2 Funnel Plot of rates of general hospital suicide per patient year |
|  |
| SM 3.3 Funnel Plot of the proportion of general hospital suicides by jumping. |
